# Epigenetic effects of casein-derived opioid peptides in SH-SY5Y human neuroblastoma cells

**DOI:** 10.1186/s12986-015-0050-1

**Published:** 2015-12-09

**Authors:** Malav S. Trivedi, Nathaniel W. Hodgson, Stephen J. Walker, Geert Trooskens, Vineeth Nair, Richard C. Deth

**Affiliations:** Department of Pharmaceutical Sciences, Nova Southeastern University, Rm # 3103, HPD building, Fort Lauderdale, FL USA; Department of Molecular and Cellular Biology, Harvard Medical School, Boston, MA USA; Wake Forest Institute for Regenerative Medicine, Wake Forest University Health Sciences, Winston Salem, NC USA; Department of Mathematical Modelling, Statistics and Bioinformatics, University of Ghent, Ghent, Belgium

**Keywords:** Epigenetics, Gluten free casein free diet, Autism, Glutathione, Gastrointestinal, Inflammation

## Abstract

**Background:**

Casein-free, gluten-free diets have been reported to mitigate some of the inflammatory gastrointestinal and behavioral traits associated with autism, but the mechanism for this palliative effect has not been elucidated. We recently showed that the opioid peptide beta-casomorphin-7, derived from bovine (bBCM7) milk, decreases cysteine uptake, lowers levels of the antioxidant glutathione (GSH) and decreases the methyl donor S-adenosylmethionine (SAM) in both Caco-2 human GI epithelial cells and SH-SY5Y human neuroblastoma cells. While human breast milk can also release a similar peptide (hBCM-7), the bBCM7 and hBCM-7 vary greatly in potency; as the bBCM-7 is highly potent and similar to morphine in it's effects. Since SAM is required for DNA methylation, we wanted to further investigate the epigenetic effects of these food-derived opioid peptides. In the current study the main objective was to characterize functional pathways and key genes responding to DNA methylation effects of food-derived opioid peptides.

**Methods:**

SH-SY5Y neuroblastoma cells were treated with 1 μM hBCM7 and bBCM7 and RNA and DNA were isolated after 4 h with or without treatment. Transcriptional changes were assessed using a microarray approach and CpG methylation status was analyzed at 450,000 CpG sites. Functional implications from both endpoints were evaluated via Ingenuity Pathway Analysis 4.0 and KEGG pathway analysis was performed to identify biological interactions between transcripts that were significantly altered at DNA methylation or transcriptional levels (*p* < 0.05, FDR <0.1).

**Results:**

Here we show that hBCM7 and bBCM7, as well as morphine, cause epigenetic changes affecting gene pathways related to gastrointestinal disease and inflammation. These epigenetic consequences exhibited the same potency order as opiate inhibition of cysteine uptake insofar as hBCM7 was less potent than bBCM7, which was less potent than morphine.

**Conclusion:**

Our findings indicate that epigenetic effects of milk-derived opiate peptides may contribute to GI dysfunction and inflammation in sensitive individuals. While the current study was performed using SH-SY5Y neuronal cellular models, similar actions on other cells types might combine to cause symptoms of intolerance. These actions may provide a potential contributing mechanism for the beneficial effects of a casein-free diet in alleviating gastrointestinal symptoms in neurological conditions including autism and other conditions. Lastly, our study also contributes to the evolving awareness of a “gut-brain connection”.

**Electronic supplementary material:**

The online version of this article (doi:10.1186/s12986-015-0050-1) contains supplementary material, which is available to authorized users.

## Background

During early development, human breast milk and bovine milk-based formulas are the best and frequently the only source of infant nutrition. Casein, a class of proteins that makes up roughly 80 % of the protein found in milk, is broken down during digestion into a number of smaller peptides. One of these breakdown products is the opioid-like peptide beta-casomorphin-7 (BCM7) [1, 2], and a structurally similar opioid-like peptide is released during digestion of wheat-derived gluten [3, 4]. Canonically, food-derived opioid peptides are reported to act on the μ-opioid receptor (μ-OR) and they are able to reach the bloodstream [5] and cerebrospinal fluid [6] with the potential to exert effects in the brain [7]. However, the downstream effects of this food-derived opioid peptide exposure, especially in terms of gene expression and regulation, are still largely unknown. Beta-casein variants are of two major sub-types; namely A1 and A2 type of beta-casein. It is noteworthy to mention that BCM-7, the opioid peptide is only derived from A1-type of beta-casein and not A2 upon digestion.

We recently showed that casein and gluten-derived opioid peptides decrease excitatory amino acid transporter 3 (EAAT3)-mediated cysteine uptake by human GI epithelial cells and human neuronal cells in a dose-dependent manner, resulting in redox and epigenetic consequences via their capacity for μ-OR activation [8]. Epigenetic regulation of gene expression is of particular importance during early development when changes in DNA or histone methylation status can exert lifelong and even transgenerational effects [9–11]. Thus milk-derived opioid peptides may exert an important epigenetic influence with extended consequences. It is noteworthy to mention that the human form of BCM-7 was far less potent as compared to the bovine form of BCM-7 in inhibition of cysteine uptake, inducing oxidative stress as well as decreased SAM levels. 

With regard to neural development, epigenetic regulation can influence neural stem cell differentiation as well as neuronal plasticity and/or neuronal maturation, which are particularly prominent during fetal and early postnatal development [12]. We previously highlighted the critical role of prenatal and postnatal epigenetic programming in the redox-based linkage between GI, brain, and immune systems, which could potentially contribute to the etiology of autism and other disorders [10]. These redox-based metabolic and epigenetic changes have not only been identified in early developmental and neurological diseases [13–15], but also characterized in diseases arising across the lifespan, including immunological [10, 16] and neurodegenerative disorders [17–19].

A gluten-free (GF) casein-free (CF) diet has been reported to improve intestinal, autoimmune and neurological symptoms in celiac disease [20, 21], autism [22] and schizophrenia [23, 24]. However, the mechanism by which a GF/CF diet improves these symptoms is not fully understood, and in the absence of definitive evidence, the potential benefit of these dietary interventions remains unexplained or considered to be poor [25]. In the current study we investigated the functional effects of milk-derived opioid peptides using pathway analysis of genome wide changes in global DNA methylation patterns, measured via methyl-CpG binding domain (MBD) protein-enriched genome sequencing (MBD-seq) in cultured human neuroblastoma cells, as well as changes in transcription status using a genome-wide microarray expression assay. The observed changes may contribute to adverse effects of milk-derived opioid peptides in sensitive individuals.

## Methods

### Materials

Morphine was obtained from Sigma Chemicals (Catalog# M8777, St. Louis, MO). Human and bovine forms of BCM-7 were custom synthesized by Neopeptide (Cambridge, MA).

### Cell culture

SH-SY5Y human neuroblastoma cells were purchased from ATCC® (Manassas, VA). Cells were grown as proliferative monolayers in 10 cm standard tissue culture dishes, containing 10 mL of alpha-modified Minimum Essential Medium (α-MEM) from Mediatech (Manassas, VA) supplemented with 1 % penicillin-streptomycin-fungizone, also from Mediatech, and 10 % fetal bovine serum (FBS) from HyClone (Logan, UT) at 37 °C with 5 % CO_2_. Cells (Passage # 4) treated for 4 h with 1 μM hBCM7, bBCM7, morphine or left untreated as a control prior to RNA or DNA extraction. This concentration was chosen on the basis of previous dose–response studies indicating that 1 μM produced maximum inhibition of EAAT3-mediated cysteine uptake.

### DNA isolation

DNA from cell culture for the analysis of DNA methylation was isolated using the FitAmp™ Blood & Cultured Cell DNA Extraction Kit from Epigentek (Farmingdale, NY). Isolated DNA was quantified using a ND-1000 NanoDrop (Wilmington, DE) spectrophotometer.

### RNA isolation

RNA from cell culture for the analysis of RNA transcription was isolated using the RNAqueous®-4PCR kit from Ambion (Austin, TX). Isolated RNA was treated with DNase, followed by RNA quantification using a ND-1000 NanoDrop spectrophotometer.

### Site-specific CpG methylation: fragmentation and MBD-capture

Genomic DNA was extracted from samples with the Easy DNA kit (Invitrogen K1800-01; Grand Island, NY) using the appropriate protocol for cell lines. DNA methylation measurement was performed using the MethylCap-Seq protocol, as described by De Meyer [26]. We used EdgeR, developed by Robinson et al. [27], for the detection of regions with differential MBD coverage between conditions. The technical and biological variation has to be estimated and modelled, while considering the discrete nature of the count values. The EdgeR package provides functions to estimate the biological variability from low number of replicates and models the count data using negative binomial distribution. We used a common dispersion estimate to obtain Differential promoter methylation test *p*-values. The region was called “differentially methylated” if the FDR adjusted *p*-value (i.e. *q*-value) was less than 0.10.

### Microarray assays

For microarray hybridizations, 500 ng of total RNA from each sample was labeled with fluorescent dye (Cy3; Amersham Biosciences Corp, Piscataway, NJ) using the Low RNA Input Linear Amplification Labeling kit (Agilent Technologies, Palo Alto, CA) following the manufacturer’s protocol. The amount and quality of the fluorescently labeled cRNA was assessed using a NanoDrop ND-1000 spectrophotometer and an Agilent Bioanalyzer. According to manufacturer’s specifications, 1.6 mg of Cy3-labeled cRNA was hybridized to the Agilent Human Whole Genome Oligo Microarray (Agilent Technologies, Inc., Palo Alto, CA) for 17 h, prior to washing and scanning. Data was extracted from scanned images using Feature Extraction Software (Agilent Technologies, Inc., Palo Alto, CA).

### Gene ontology and pathway analysis

Pairwise comparisons (e.g. hBCM-7 [4 h] vs bBCM-7 [4 h]) were carried out using Student’s *t*-test (at a fold change ≥ 1.5, *raw p* ≤ 0.05; the N values in this pilot study were small such that applying a Benjamini & Hochberg FDR correction resulted in no DETs for the hBCM-7 and bBCM-7 versus control comparisons) to generate lists of differentially expressed genes. For each comparison between treatments, individual lists of differentially expressed (and differentially methylated) genes were uploaded to the Ingenuity® Pathway Analysis (IPA) program (http://www.ingenuity.com) for determination of biologically relevant functional categories (gene ontologies) and canonical pathway involvement. The significance value associated with Functional Analysis for a dataset is a measure of the likelihood that the association between a set of Functional Analysis molecules in these data and a given process or pathway is due to random chance. The *p*-value is calculated using the right-tailed Fisher Exact Test. In general, *p*-values less than 0.05 indicate a statistically significant, non-random association.

### Hierarchical clustering

Clustering of samples was performed using the Euclidean metric and data where each variable has been normalized to mean = 0 and variance = 1, using linkage criteria set to ‘average linkage’. The resulting heat maps display the top 50 differentially-expressed transcripts compared to pooled RNA from control cells (*p* ≤ 0.001). The list of probes is found in Additional file 1: Table S1.

### Statistical analysis

Statistical analyses were carried out using Graph Pad Prism® version 5.01. Student’s *t*-test for independent means was used to test for significant differences between untreated control and experimental groups. Data were expressed as mean ± standard error of the mean (SEM). Comparisons between multiple groups of data were conducted using one-way analysis of variance (ANOVA) followed by Tukey’s post-hoc test was used to determine the differences between individual groups.

## Results

Our previous results reported global DNA methylation changes under the influence of opioid peptides. However, the hBCM-7 was far less potent as compared to the bovine form of BCM-7 in altering the global DNA methylation status as well as changes in GSH and SAM levels [9]. In the current study, we investigated the genome-wide epigenetic changes under the influence of these opioid peptides. To investigate functional pathway and gene network changes induced by hBCM7, bBCM7 and morphine, the DNA methylation MBD-seq and DNA microarray data were collected. For this purpose, control SH-SY5Y neuroblastoma cells and cells treated for 4 h with 1 μM hBCM7, bBCM7 or morphine were used, the latter serving as a positive opioid effect control. The dose and time-points were selected based on our previous studies [8]. Whole genome DNA MBD-seq revealed differentially methylated promoter transcripts (DMTs), as defined by FDR < 0.1 and microarray data revealed differentially expressed transcripts (DETs), defined by fold change ≥ 1.5 and raw *p*-value ≤ 0.05, which included differentially methylated/ transcribed genes from both genic and non-coding regions.

**Fig. 1:**
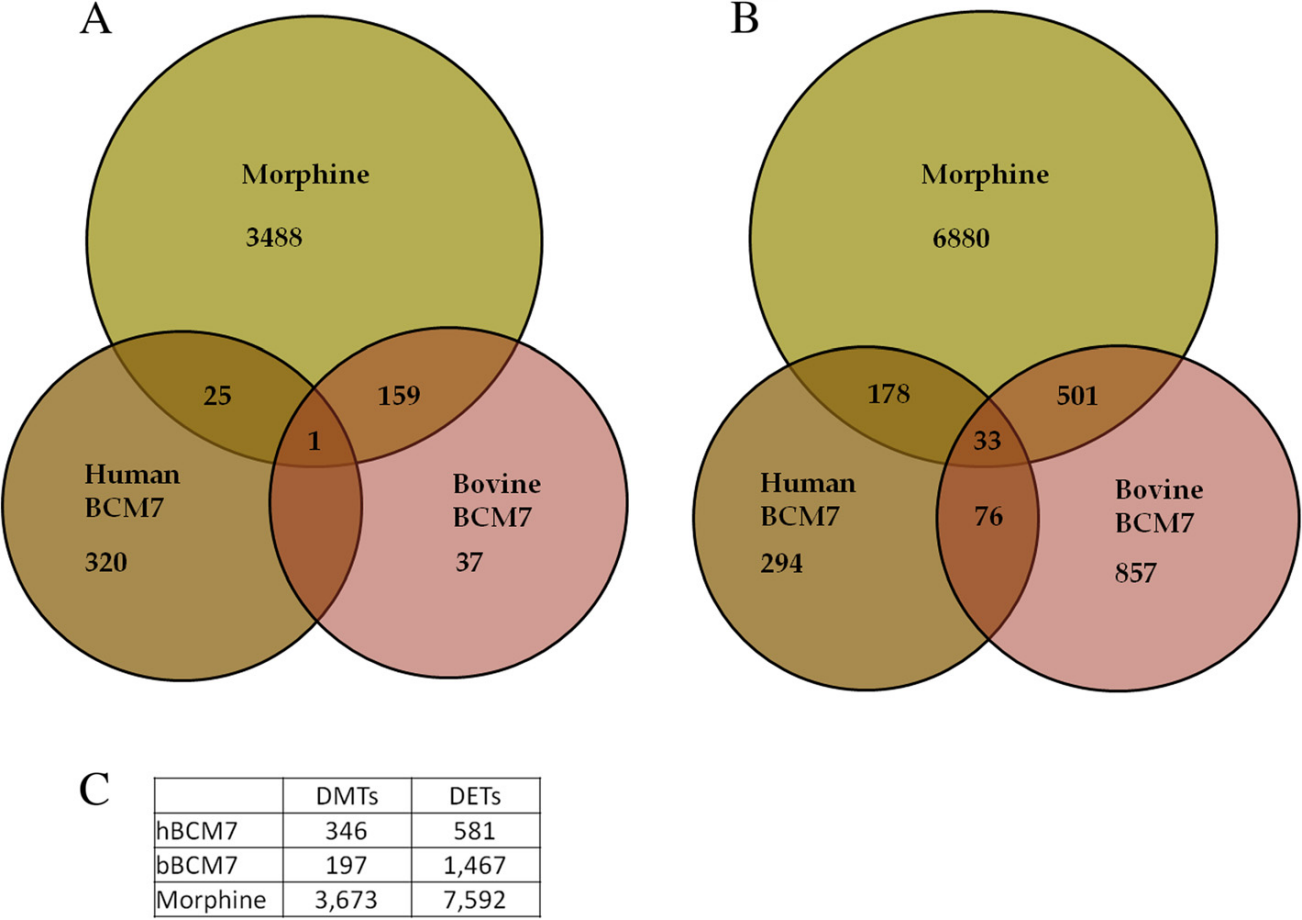
Venn diagrams representing overlap in DETs and DMTs between treatment conditions. SH-SY5Y human neuroblastoma cells were treated with 1 μM morphine, bBCM7 or hBCM7 for 4 h (*n* = 5). Gene expression was analyzed by genome-wide microarray to generate lists of differentially expressed transcripts (DETs; Panel **a** and DNA methylation was analyzed by MBD-seq to yield lists of differentially methylated transcripts (DMTs; Panel **b** DMTs and DETs were plotted to illustrate overlapping transcript changes caused by one or more of the treatment groups compared with non-treated control. For DETs, *N* = 3; fold change ≥ 1.5; raw *p* ≤ 0.05. For DMTs, *N* = 5, FDR < 0.1

Treatment with the prototypical opiod morphine (positive control) resulted in 4950 DETs, while bBCM7 treatment yielded 1467 and hBCM7 treatment resulted in 581; hence the rank order in gene expression changes was morphine > bBCM7 > hBCM7 (Fig. 1). Morphine treatment resulted in a distinct bias towards gene up-regulation, with 4785/4950 DETs (96.7 %) reflecting increased expression, while for bBCM7 1259/1467 (85.8 %) of DETs were associated with increased expression. By contrast, only 209/581 DETs (36.0 %) for hBCM7 treatment reflected up-regulation.

**Fig. 2:**
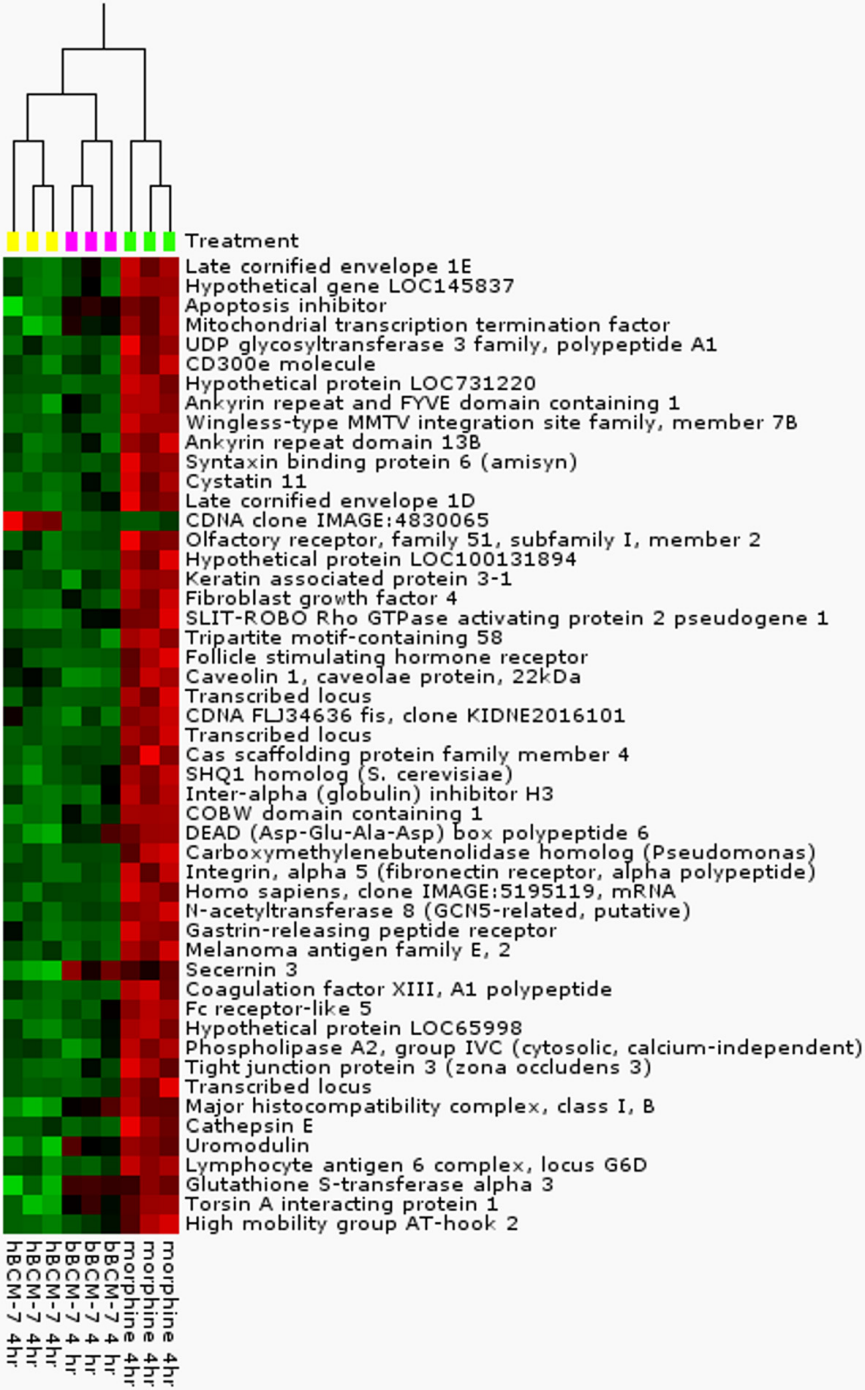
Differential gene expression in SH-SY5Y human neuroblastoma cells treated with morphine, bBCM7 or hBCM7. Gene expression data were obtained using Agilent Human Whole Genome Oligo Microarray. The heat map represents the top 50 differentially expressed probes compared to control (*p* < 0.001). Each column represents a single treatment and each row represents a single gene. At the top of the diagram is a dendogram that displays the relatedness of the individual samples

Next, about 501 DETs were shared by both morphine and bBCM7, 178 overlapped between morphine and hBCM7, 76 overlapped between hBCM7 and bBCM7, while 33 overlapped between all three treatments. Morphine and bBCM7 shared a higher number of DETs, as compared to morphine and hBCM7 (Fig. 1). Hierarchical clustering for top 50 DETs showed that changes in gene expression for hBCM7 and bBCM7 are more related to each other than to morphine (Fig. 2).

Morphine treatment resulted in 3673 promoter region DMTs (defined as transcription start site ± 500 bp), bBCM7 ~ 197 and hBCM7 ~ 346 DMTs; with the rank for differential promoter region methylation as morphine > hBCM7 > bBCM7. Of these DMTs, morphine and bBCM7 shared 159, morphine and hBCM7 had 25 common and only 1 DMTs overlapped between all three treatments (Fig. 1).

**Fig. 3:**
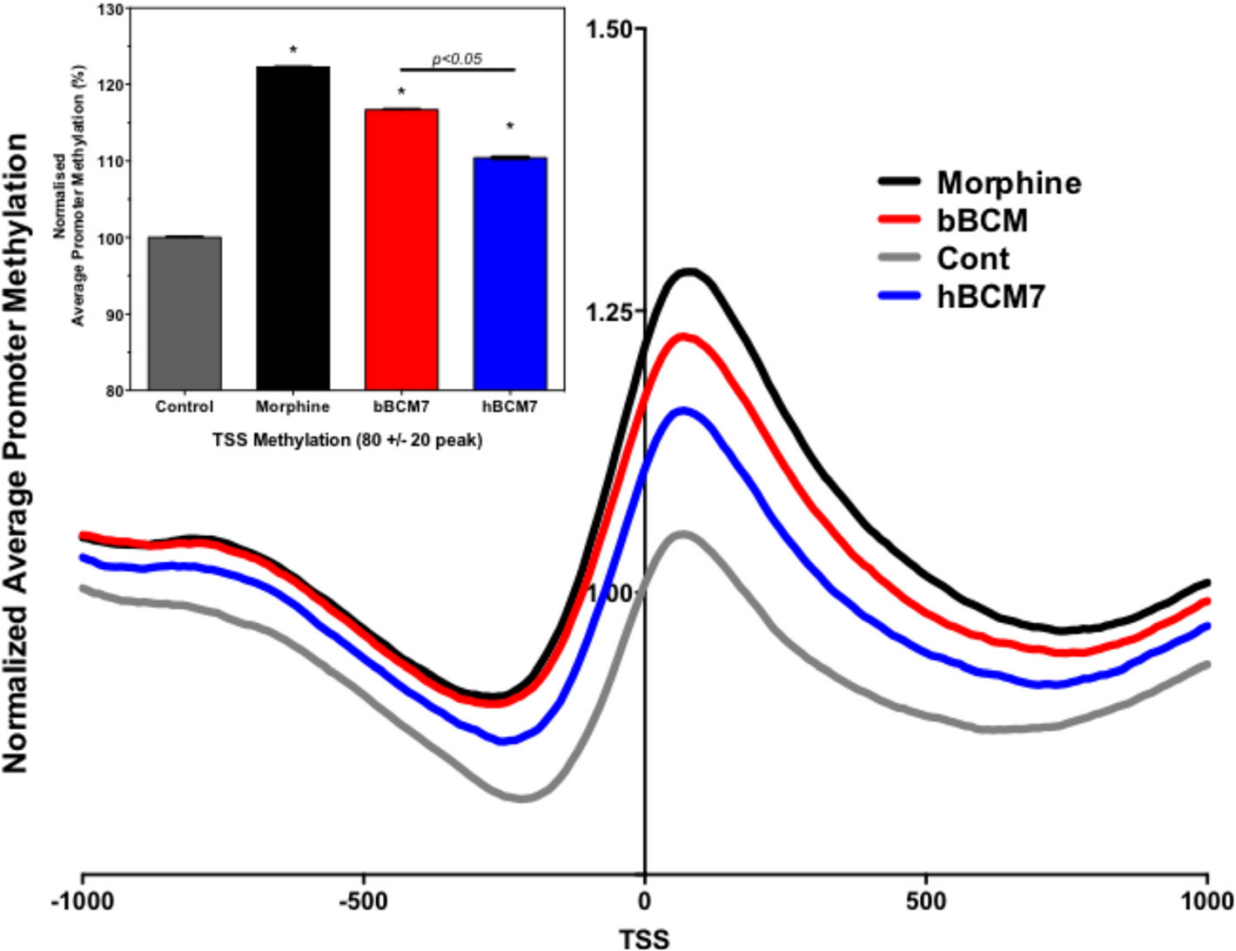
Methylation profiles in control and treated cells. SH-SY5Y human neuroblastoma cells were treated with 1 μM morphine, bBCM7 or hBCM7 for 4 h (*n* = 5) and DNA methylation was analyzed by MBD-seq. 53,561 genes were aligned at their transcription start site (TSS) and average methylation between -3000 bp and +3000 bp was computed and normalized to values at -3000 bp

Next, promoter region methylation of all 53,561 genes was evaluated at their transcription start sites (TSS) and relative DNA methylation was calculated for 1000 bp in either direction, normalized to the level at the control at TSS. A fluctuating methylation density was observed in the TSS region in untreated control cells, featuring a peak of increased methylation at +65 to +80, flanked by two valleys of decreased methylation at −200 to −300 and +600 to +800 bp (Fig. 3). While the overall pattern of TSS DNA methylation was unchanged, a 4 h opioid treatment increased genome-wide TSS methylation. Morphine caused a 22 % increase in DNA methylation above the control level at the + 65 to +80 peak, followed by bBCM7 (17 %) and hBCM7 (11 %); (Inset in Fig. 3). The relative efficacy of these opioids to alter DNA promoter methylation mirrors their efficacy in their inhibition of cysteine uptake as previously reported by this lab, which also had an efficacy order of morphine > bBCM7 > hBCM7 [28]. Thus bBCM7 exhibits a higher efficacy for increasing TSS methylation than hBCM7 (*p* < 0.05).

Using ingenuity pathway analysis (IPA), we examined the biological interactions between genes and noncoding mRNAs, which changed in either their promoter region methylation status or their transcription levels upon exposure to hBCM7, bBCM7 or morphine. The functional biological pathways most significantly populated with elements from these lists were identified (Additional file 1: Table S2). Among the diseases and disorders that were found to be significantly associated with the observed DETs and DMTs, three diseases or disorders of particular interest were identified: gastrointestinal disease, inflammatory disease and inflammatory response (Table 1). Morphine-induced DMTs were associated with gastrointestinal disease and inflammatory response, while morphine induced DETs were associated with inflammatory disease and response. hBCM7-induced DMTs were associated with gastrointestinal disease, and bBCM7 DMTs were associated with inflammatory disease.

**Table 1:** SH-SY5Y human neuroblastoma cells were treated with 1 μM morphine, bBCM7 or hBCM7 for 4 h (*n* = 5). DNA methylation was measured by MBD-seq, while mRNA transcription was measured with whole genome microarray. IPA functional analysis was performed with DETs and DMTs for the treatments vs. control. *P* values were generated by IPA for gene ontology categories associated with gastrointestinal disease, inflammatory disease or inflammatory response for each treatment vs. control. *P*-values are displayed as a range based on the number of genes from the experimental list that appears in a particular ontology [sub-group] within a Disease or Disorder category

Disease or disorder	*P*-value ranges for DMTs		
	Morphine	hBCM7	bBCM7
Gastrointestinal disease	9.34E-08 – 1.07E-02	1.09E-12 – 7.32E-03	
Inflammatory disease			3.96E-03 – 4.51E-02
Inflammatory response	5.46E-05 – 1.07E-02		
	*P*-value ranges for DETs		
	Morphine	hBCM7	bBCM7
Gastrointestinal disease			
Inflammatory disease	1.33E-12 – 1.35E-03		
Inflammatory response	4.06E-08 – 1.33E-03		

**Fig. 4:**
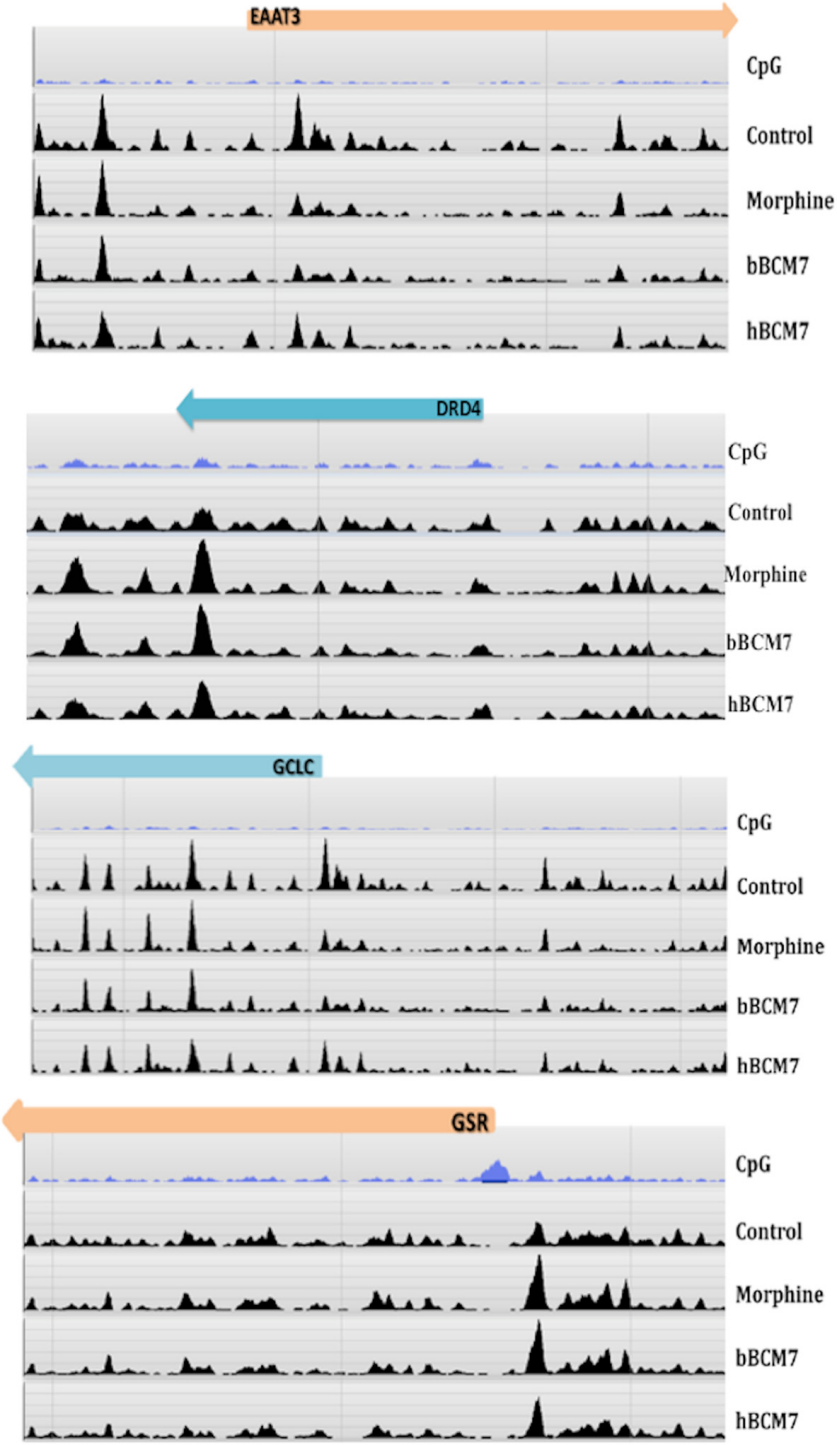
DNA-methylation distribution of genes involved in transsulfuration pathway. Gene-specific graphical representation of distribution of methylation peaks for the genes involved in the transsulfuration pathway. The Refseq genes are shown in orange / blue color. The CpG density for the specific region of the gene is also indicated. Reference genome is from the H2G2 genome browser which uses the hg19 version of the human genome

Lastly, we investigated if there were any underlying opioid-mediated changes in the epigenetic status of enzymes and proteins involved in the transulfuration and glutathione synthesis pathway. We observed differences in DNA methylation levels of several genes from the transsulfuration pathway and the methionine cycle (Fig. 4). Both genic and intragenic methylation changes were observed. More importantly these changes observed in the epigenetic patterns were also correlated the gene expression changes we reported in our previous studies. For example, morphine and bBCM7 both induced elevated levels in the DNA methylation patterns in the promoter region of the gene coding for the excitatory amino acid transporter (EAAT3). This corresponded with our previous results wherein we reported that morphine and bBCM7 treatment was associated with decreased levels of EAAT3 transporter as measured using qPCR [8]. Similar association between changes in mRNA levels and the DNA methylation levels were also observed with other enzymes and proteins involved in the transulfuration pathway [8]. Thus, morphine, hBCM7 and bBCM7 produce changes in DNA methylation, which may affect metabolic pathways regulating GSH and SAM levels.

## Discussion

Epigenetic regulation not only allows a pluripotent cell to differentiate into alternative cell types containing the same genetic information, but it also provides a mechanism for plastic and adaptive responses to changes in the cellular environment arising from physiological substances, xenobiotics, or food, representing a form of genetic metadata. We previously outlined a possible metabolic link between redox and methylation, and a potential mechanism by which changes in redox status translate into epigenetic outcomes [14, 19] and lastly, the potential for nutritional factors to affect prenatal and postnatal epigenetic programming [10]. Redox and epigenetic changes have been implicated in early developmental and neurological diseases [13–15], as well as immunological [10, 16] and neurodegenerative disorders [17–19]. Recently, we showed that redox effects of food-derived peptides with opioid activity exert powerful control over DNA methylation [8]; however, a synthetic peptide constituted with a scrambled sequence of the BCM7, did not show any effect on the global DNA methylation (Additional file 2: Figure S3), acting as a negative control, not activating the mu opioid receptor and inducing downstream epigenetic changes. Thus, the current results are an extension and insightful investigation of our previous work, clarifying the epigenetic effects of food-derived opioids, especially milk-derived opioids.

Using SH-SY5Y human neuroblastoma cells, we found that bBCM7 and hBCM7, as well as the prototypical opiate agonist morphine, caused epigenetic as well as transcriptional changes that could be linked to pathways contributing to gastrointestinal disease and inflammation in the GI tract. Although, it is noteworthy to mention that the bBCM-7 released from A1-type of beta casein was far more potent in inducing these epigenetic changes as compared to the hBCM-7. It should be noted that the primary endpoint for the current study was not to model GI diseases in SH-SY5Y cells, but it is highly intriguing that the pathway analysis identified GI function and diseases to be the most highly affected. This result may not be surprising since we have previously showed that EAAT3 is present at high levels in human intestinal epithelial cells (Caco2 cells), and food-derived opioid peptides can inhibit EAAT3-mediated cysteine uptake in these cells, resulting in altered redox and methylation capacity [8]. Thus, these molecular effects of opiates on antioxidant levels and methylation status can occur in different cell types and can result in overlapping epigenetic consequences.

Several previous studies have indicated the use of SH-SY5Y cells as models for neuronal cells for research related to central nervous system [8] as well as enteric nervous system [29]. Furthermore, oxidative stress has recently been recognized as a significant factor in the pathogenesis of gastrointestinal complications [30], and treatment with GSH or the GSH released from glial cells resulted in alleviation of oxidative stress in neuronal cells [29] as well as gastrointestinal symptoms. Mu-opioid receptors are highly expressed in the gastrointestinal tract including in neurons of the enteric nervous system. Hence, although we did not use SH-SY5Y as a model for induction of gastrointestinal diseases, we observed that induction of oxidative stress resulted in epigenetic changes which corresponded to gastrointestinal diseases. These results provide a potential explanation for the observed high prevalence of GI dysfunction co-morbidity in patients with neurological disorders including autism spectrum disorders.

Both wheat- and milk-derived opiate peptides inhibit the uptake of cysteine, resulting in a decrease in GSH synthesis [8]. This decrease in antioxidant capacity leads to inhibition of the redox sensitive enzyme methionine synthase and a decrease in the availability of the methyl donor S-adenosylmethionine (SAM). Oxidative stress and decreased levels of SAM and GSH have been associated with autism by multiple studies [31–41]. The decrease in SAM translates into effects on global DNA methylation, with epigenetic consequences, which can contribute to neurological and neurodevelopmental disorders such as autism [42–47].

The changes in DNA methylation and gene expression we observed were associated with inflammation and gastrointestinal disease-related metabolic pathways. One way to interpret this result is a dysfunctional positive feed-back loop in which food-derived opiate peptides decrease cysteine uptake from the GI tract, causing inflammation and gastrointestinal disease which further limits the ability to take up cysteine and other important nutrients. Avoiding milk-derived BCM-7 might be beneficial to prevent this inflammation in sensitive individuals; however, supporting clinical evidence is not yet reported and hence any clinical extrapolation of these in vitro studies should be done with caution. In some, but not all studies, dietary intervention with a gluten-free casein-free diet has been shown to improve symptom presentation in autistic subjects [22, 48, 49]. BCM7 has been linked to delayed psychomotor development in infants [50]. Interestingly, a genetic variant in dipeptidylpeptidase IV (DPP-IV), the enzyme responsible for hydrolysis of BCM7, was found to be significantly overrepresented in autistic male subjects [51], and autoantibodies against DPP-IV are found in autistic subjects [52]. Thus diminished activity of DPP-IV may contribute to increased activity of BCM7 in autism. Our findings also highlight a difference in the efficacy of human *vs.* bovine-derived BCM7 in altering DNA promoter methylation and mRNA transcription inflammation. The ability of BCM7 to alter gene expression has been previously reported [12]. Here we show that bBCM7 acts more like morphine than hBCM7, in the both magnitude of DNA promoter methylation changes, a similar bias toward mRNA up-regulation and in the extent of overlapping discrete DMTs and DETs. Thus the epigenetic effects of bBCM7 may be quantitatively and qualitatively different from those of hBCM7, which could potentially also provide a molecular rationale for the recommendation of breastfeeding *vs.* formula feeding during early development.

## Conclusion

Results from the current study indicate that epigenetic effects of milk-derived opiate, especially bovine BCM-7 peptides may possibly contribute to GI dysfunction and to development of inflammation. Thus eliminating these opioid peptides from the diet may reverse these effects in sensitive individuals, although future clinical studies will be required to further investigate this mechanism. While preliminary, our findings indicate a “gut-brain” epigenetic relationship.

The data set supporting the results of this article are included as additional files for supplementary Figure S1, S2 and Additional file 2: Figure S3 on the journal website.

## Additional files

Additional file 1: Table S1. List of 50 differentially transcribed probes from heat maps used in figure 2. (PDF 66.6 kb) Additional file 2: Table S2. Diseases and disorders generated by IPA analysis of DMTs and DETs corresponding to treatment. The p-value range listed represents the range of significant (P < 0.05) pathways associated with the listed disease or disorder. The number of probes indicates the number of differentially methylated or transcribed transcripts associated with the disease or disorder. (PDF 60.5 kb)

## Abbreviations

bBCM7: bovine isoform of BCM7
DETs: differentially expressed transcripts.
DMTs: differentially methylated transcripts.
EAAT3: excitatory amino acid transporter type 3
GF/CF: gluten free/ casein free
GI: gastrointestinal
GSH: glutathione
hBCM7: human isoform of BCM7
SAM: S-adneosylmethionine
