# Supplementary material for: Epigenetic effects of casein-derived opioid peptides in SH-SY5Y human neuroblastoma cells
Source: Nutr Metab (Lond). 2015 Dec 9;12:54. doi: 10.1186/s12986-015-0050-1 (PMC4673759; doi:10.1186/s12986-015-0050-1)
Supplement: Additional file 1: Table S1. — List of 50 differentially transcribed probes from heat maps used in figure 2. (PDF 66.6 kb) [file 12986_2015_50_MOESM1_ESM.pdf]

	Probe
1	Late cornified envelope 1E
2	Hypothetical gene LOC145837
3	Apoptosis inhibitor
4	Mitochondrial transcription termination factor
5	UDP glycosyltransferase 3 family, polypeptide A1
6	CD300e molecule
7	Hypothetical protein LOC731220
8	Ankyrin repeat and FYVE domain containing 1
9	Wingless-type MMTV integration site family, member 7B
10	Ankyrin repeat domain 13B
11	Syntaxin binding protein 6 (amisyn)
12	Cystatin 11
13	Late cornified envelope 1D
14	CDNA clone IMAGE:4830065
15	Olfactory receptor, family 51, subfamily I, member 2
16	Hypothetical protein LOC100131894
17	Keratin associated protein 3-1
18	Fibroblast growth factor 4
19	SLIT-ROBO Rho GTPase activating protein 2 pseudogene 1
20	Tripartite motif-containing 58
21	Follicle stimulation hormone receptor
22	Caveolin 1, caveolae protein, 22kDa
23	Transcribed locus
24	CDNA FI_J34636 fis, clone KIDNE2016101
25	Transcribed locus
26	Cas scaffolding protein family member 4
27	SHQ1 homolog (S. cerevisiae)
28	Inter-alpha (globulin) inhibitor H3
29	COBW domain containing 1
30	DEAD (Asp-Glu-Ala-Asp) box polypeptide 6
31	Carboxymethylenebutenolidase homolog (Pseudomonas)
32	Integrin, alpha 5 (fibronectin receptor, alpha polypeptide)
33	Homo sapiens, clone IMAGE:5195119, mRNA
34	N-acetyltransferase 8 (GCN5-related, putative)
35	Gastrin-releasing peptide receptor
36	Melanoma antigen family E, 2
37	Secernin 3
38	Coagulation factor XIII, A1 polypeptide
39	Fc receptor-like 5
40	Hypothetical protein LOC65998
41	Phospholipase A2, group IVC (cytosolic, calcium-independent)
42	Tight junction protein 3 (zona occludens 3)
43	Transcribed locus
44	Major histocompatibility complex, class I, B
45	Cathepsin E
46	Uromodulin
47	Lymphocyte antigen 6 complex, locus G6D
48	Glutathione S-transferase alpha 3
49	Torsin A interacting protein 1
50	High mobility group AT-hook 2

Supplemental Table 1. List of 50 differentially transcribed probes from heat maps used in figure 2.
