# Supplementary material for: Epigenetic effects of casein-derived opioid peptides in SH-SY5Y human neuroblastoma cells
Source: Nutr Metab (Lond). 2015 Dec 9;12:54. doi: 10.1186/s12986-015-0050-1 (PMC4673759; doi:10.1186/s12986-015-0050-1)
Supplement: Additional file 2: Table S2. — Diseases and disorders generated by IPA analysis of DMTs and DETs corresponding to treatment. The p-value range listed represents the range of significant (P < 0.05) pathways associated with the listed disease or disorder. The number of probes indicates the number of differentially methylated or transcribed transcripts associated with the disease or disorder. (PDF 60.5 kb) [file 12986_2015_50_MOESM2_ESM.pdf]

Methylation		
Disease or Disorder	P-value Ranges	Probes
Morphine		
Cancer	8.28E-24 - 1.10E-02	3020
Gastrointestinal Disease	9.34E-08 - 1.07E-02	1682
Reproductive System Disease	1.59E-06 - 6.94E-03	747
Inflammatory Response	5.46E-05 - 1.07E-02	435
Respiratory Disease	5.61E-05 - 1.10E-02	354
hBCM7		
Cancer	4.50E-25 - 8.75E-03	1770
Gastrointestinal Disease	1.09E-12 - 7.32E-03	1212
Metabolic Disease	7.24E-06 - 5.57E-03	312
Reproductive System Disease	2.33E-05 - 5.43E-03	417
Endocrine System Disorders	6.01E-05 - 5.43E-03	390
bBCM7		
Neurological Disease	3.40E-03 - 4.07E-02	14
Organismal Injury and Abnormalities	3.40E-03 - 3.82E-02	11
Connective Tissue Disorders	3.96E-03 - 4.07E-02	6
Dermatological Diseases and Conditions	3.96E-03 - 4.51E-02	10
Inflammatory Disease	3.96E-03 - 4.51E-02	9
Transcription		
Disease or Disorder	P-value Ranges	Probes
Morphine		
Inflammatory Response	1.33E-12 - 1.35E-03	274
Connective Tissue Disorders	4.06E-08 - 1.33E-03	171
Inflammatory Disease	4.06E-08 - 1.33E-03	223
Skeletal and Muscular Disorders	4.06E-08 - 1.33E-03	174
Nutritional Disease	3.19E-07 - 3.59E-04	88
hBCM7		
Developmental Disorder	3.52E-05 - 1.14E-02	28
Cardiovascular Disease	4.61E-05 - 1.14E-02	23
Skeletal and Muscular Disorders	6.28E-05 - 1.14E-02	37
Cancer	6.95E-05 - 1.14E-02	69
Organismal Injury and Abnormalities	6.95E-05 - 1.14E-02	33
bBCM7		
Cancer	1.27E-04 - 3.28E-02	185
Reproductive System Disease	1.27E-04 - 3.01E-02	19
Endocrine System Disorders	2.03E-04 - 3.28E-02	22
Organismal Injury and Abnormalities	3.03E-04 - 3.28E-02	51
Infectious Disease	1.08E-03 - 3.28E-02	18

Supplemental Table 2. Diseases and disorders generated by IPA analysis of DMTs and DETs corresponding to treatment. The p-value range listed represents the range of significant ($P < 0.05$) pathways associated with the listed disease or disorder. The number of probes indicates the number of differentially methylated or transcribed transcripts associated with the disease or disorder.
